# Rehabilitation of facial palsy by the lengthening temporalis myoplastie: A case report

**DOI:** 10.1016/j.amsu.2020.12.050

**Published:** 2021-01-05

**Authors:** Ouassime Kerdoud, Rachid Aloua, Faiçal Slimani

**Affiliations:** a Faculty of Medicine and Pharmacy, Hassan II University of Casablanca, B.P 5696, Casablanca, Morocco; b Oral and Maxillofacial Surgery Department, CHU Ibn Rochd, B.P 2698, Casablanca, Morocco

**Keywords:** Dynamic reanimation, Facial palsy, Lengthening temporalis myoplasty, Transfer

## Abstract

**Introduction:**

The lengthening temporalis myoplasty (LTM) is defined as a transfer of the entire temporal muscle from the coronoid process to the labial commissure reinserted into the orbicularis muscle.

**Presentation of case:**

a 60-year-old man with grade III longstanding facial paralysis of the right hemi-face secondary to a right total parotidectomy. The surgery was performed for the rehabilitation of the right hemi-facial side by the lengthening temporalis myoplasty. The follow-up was favorable with improvement of the facial dynamics.

**Discussion:**

Surgical management of the longstanding facial palsy is a real challenge. The lengthening temporalis myoplasty offers several advantages; technically is a simple. This technique was demonstrated in severe neglected facial palsy and is performed when there is a definitive complete, or almost complete, loss of the facial nerve and the trigeminal is preserved. Effective rehabilitation through training and physical therapy is necessary to optimize results.

**Conclusion:**

Facial palsy should no longer be permanent, surgical techniques as lengthening temporalis myoplasty with early postoperative physiotherapy leads to good results. Preoperative planning and early recognition of issues can avoid postoperative complications.

## Highlights

• The value and contribution of temporal muscle lengthening myoplasty as a surgical technique in the long neglected facial palsy.
• Longstanding Facial palsy should no longer be permanent.
• The main treatment of surgical management of neglected facial plasy is to achieve a satisfactory functional and aesthetic results.
• Effective rehabilitation through training and physical therapy is necessary to optimize results.

## 1 Introduction

The lengthening temporalis myoplasty (LTM) is defined as a transfer of the entire temporal muscle from the coronoid process to the labial commissure reinserted into the orbicularis muscle [1]. This surgical technique makes it possible to regain a spontaneous smile in the context of long neglected facial paralysis.

Moreover, the temporal muscle (masticatory muscle) functions after the transfer and rehabilitation are integrated into the cortical pattern of the smile.

## 2 Case presentation

Our work is a single case report and has been reported in line with the SCARE criteria [[2], [3]].

A 60-year-old man with the following history: operated for a right parotid tumor by ENT department in 1998 and in 2010 complicated by sequelae of facial palsy. He received immediately a short-term post-operative corticosteroid therapy and ophthalmic eye drops based on artificial tears. He was referred to our department's consultation for specialized care. No other personal or family history was raised during the patient interrogation. He complained of a longstanding facial palsy of the right hemi-face, unable to lift the side of his mouth.

**Fig. 1:**
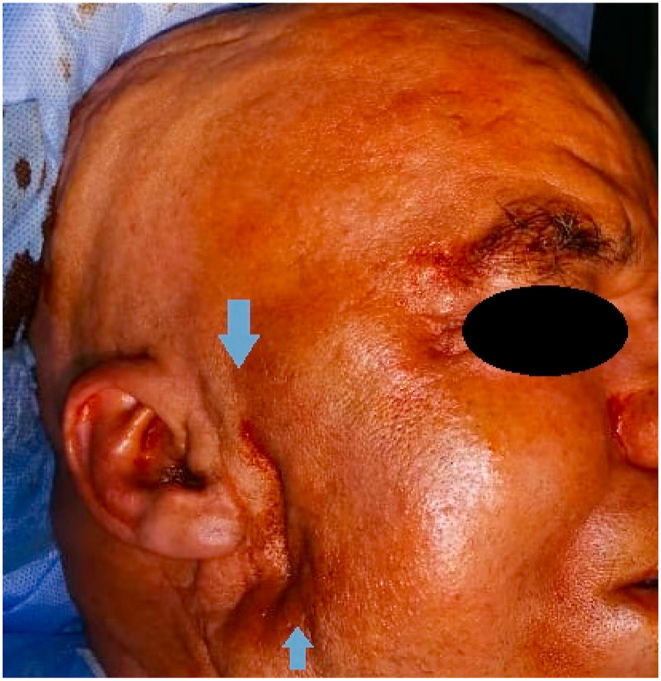
Depression facing the right parotid region.

**Fig. 2:**
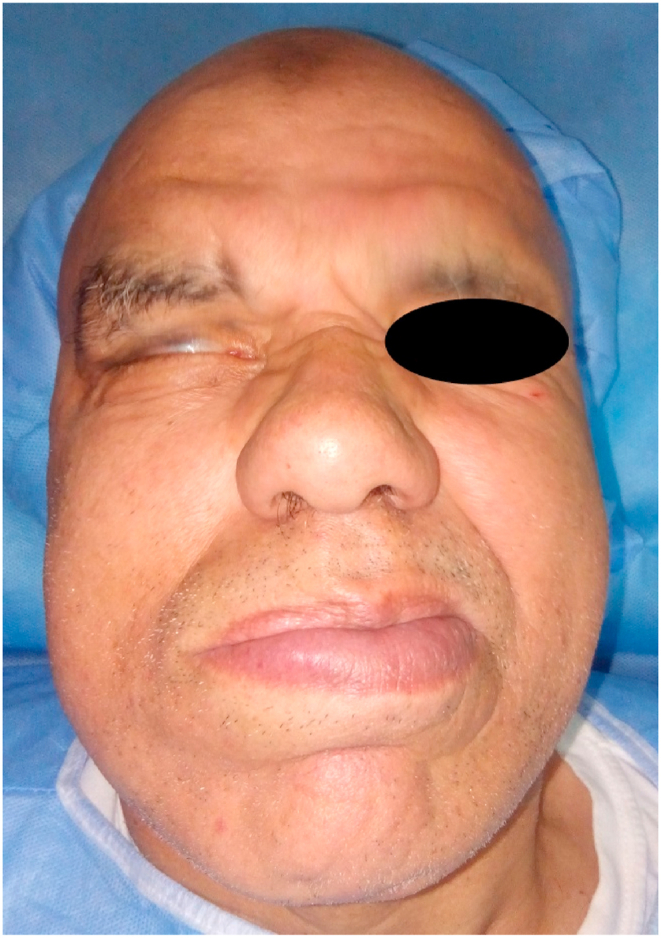
Facial paralysis Grade III.

Initial physical examination showed a depression in the right parotid region (Fig. 1), grade III facial paralysis with a drooping right labial commissure, erasure of the right naso-labial fold and right frontal wrinkles, attraction of the mouth on the healthy side with inability to whistle, incomplete closure of the eyelashes on the affected side, also we noted the blink reflex during conversation (Fig. 2).

The electroneuromyogram (ENMG) showed a neuro-praxis of the right facial nerve with decreased amplitudes and no signs of activity at rest.

**Fig. 3:**
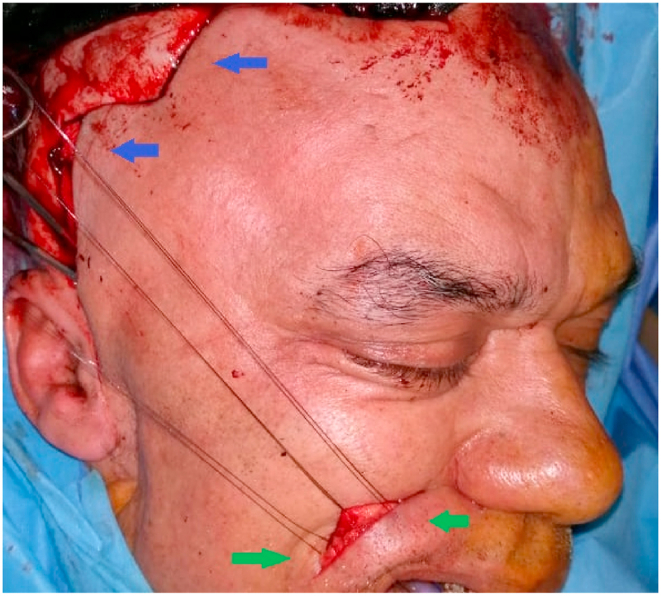
Intraoperative image: hemi-coronal and naso-labial approach (tuning and suturing). The patient received antibiotics and antalgics daily for 10 days.

Based on the positive medical history and clinical examination, surgery was indicated and performed by the chief professor of our department who has 15 years of operative experience. The surgical procedure had the aim of resuscitating the labial commissure by lengthening temporalis myoplasty (Fig. 3). In a first step in the temporal region: A hemi-coronal incision was made with sub-galeal detachment and a release of the temporal muscle was performed. Second, at the labial commissure, a naso-labial approach was performed, osteotomy of the coronoid process with tunneling and recovery of the temporal muscle, followed by suture of the temporal muscle tendon with the orbicularis muscle and the modiolus.

On the seven day, the scar was clean and non-inflammatory with no subgaleal hematoma and the patient started facial physiotherapy after the 3rd week.

Postoperative periods were favorable with complete recovery at the 30-day follow-up visit.

Routine follows up 3, 6, and 12 months later showed an improvement of facial dynamics.

## 3 Discussion

The lengthening temporalis myoplasty (LTM) was described by Daniel Labbé in 1997 as a modification of McLaughlin's and H. Gillies' temporal myoplasty [[4], [5], [6]].

The lengthening temporalis myoplasty is a dynamic technique that allows regaining lost movements by transferring from one mobile point to another mobile point. In the literature, many authors have remodified the technique, in particular the partial transfer of temporal muscle-tendon, which makes it possible to control the distance and direction of traction on the reanimated commissure. It has been objectively demonstrated that it is capable of restoring symmetry to the paralyzed face in a large number of patients in a single procedure [7].

In the case of prolonged facial nerve paralysis, where the facial muscles have undergone fibrosis or atrophy, the transfer of the innervated muscle seems necessary to restore an active movement integrated into the facial smile pattern [8,9].

The fixation of the tendon of the temporal muscle, which has been achieved in this case, while respecting its width (3–4cm) at the level of the naso-labial fold, makes it possible to reproduce the mirror effect in relation to the healthy side [10].

Therefore, this surgical technique is performed when there is a definitive complete, or almost complete, loss of the facial nerve and the trigeminal is preserved. This is verified by the electromyogram, which is essential before any transfer of the temporal muscle [11].

The intraoperative smile evident during muscle electro-stimulation provides crucial information about the action vector of the temporal muscle and indicates whether the insertion of the tendon to the perioral region is secure, is located correctly and precisely, and is symmetrical to the healthy, non-paralyzed side [12].

Effective rehabilitation through training and physical therapy is necessary to optimize results.

This surgery is intended for adults and children (must be 8 years old, so that they can be actors of their rehabilitation) having:

- A complete facial paralysis (affecting all levels of the face) with no sign of clinical and electrical recovery for 18 months.
- An incomplete facial paralysis but whose rehabilitation does not allow any more recovery.

**Fig. 4:**
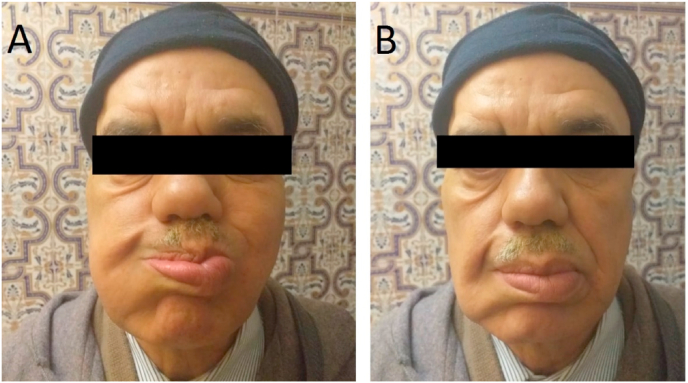
Facial dynamics after 1 month of rehabilitation (A, B).

Concerning our observation, it is a neglected facial paralysis following a total parotidectomy which is a common complication described during the parotid surgery. The delay in the management of facial paralysis has led to muscle atrophy in the right hemi-facial area, hence the justified indication for the lengthening temporalis myoplasty. Regarding the evolution and prognosis, the surgical follow-up was simple with clean and non-inflammatory scarring. Facial re-education was started from the 3rd week. At 1 month postoperatively, there was an improvement in facial kinetics in the right hemi-face with disappearance of the right cheek ptosis (Fig. 4).

## 4 Conclusion

Facial paralysis should no longer be permanent. Resuscitation of the labial commissure is only one element of the overall treatment of the paralyzed face. Surgery for peripheral facial paralysis must necessarily be accompanied by significant post-operative rehabilitation. The main treatment of surgical management of neglected facial plasy is to achieve satisfactory functional and aesthetic results.

## Provenance and peer review

Not commissioned, externally peer-reviewed.

## Ethical approval

Written informed consent was obtained from the patient for publication of this case report and accompanying images. A copy of the written consent is available for review by the Editor-in-Chief of this journal on request.

## Source of funding

The authors declared that this study has received no financial support.

## Author contribution

Ouassime kerdoud: Corresponding author writing the paper.
Rachid Aloua: writing the paper.
Faiçal Slimani: Correction of the paper.

## Registration of Research Studies

1. Name of the registry: research registry.
2. Unique Identifying number or registration ID: 6345.
3. Hyperlink to your specific registration (must be publicly accessible and will be checked):

## Guarantor

Ouassime Kerdoud.

## Declaration of competing interest

Authors of this article have no conflict or competing interests. All of the authors approved the final version of the manuscript.
